# Exploring the Environmental Resistome and Bacterial Novelty in Marine Isolates from the North Portuguese Coast

**DOI:** 10.3390/antibiotics15010110

**Published:** 2026-01-22

**Authors:** Ofélia Godinho, Olga Maria Lage, Sandra Quinteira

**Affiliations:** 1 CIMAR/CIIMAR—Interdisciplinary Centre for Marine and Environmental Research, University of Porto, 4450-208 Matosinhos, Portugal; ofeliagodinho95@gmail.com (O.G.); olga.lage@fc.up.pt (O.M.L.); 2 Department of Biology, Faculty of Sciences, University of Porto, 4169-007 Porto, Portugal; 3 CIBIO—Research Center in Biodiversity and Genetic Resources, InBIO, Research Network in Biodiversity and Evolutionary Biology, Associated Laboratory, University of Porto, Campus de Vairão, Rua Padre Armando Quintas 7, 4485-661 Vairão, Portugal; 4 BIOPOLIS Program in Genomics, Biodiversity and Land Planning, University of Porto, Campus de Vairão, Rua Padre Armando Quintas 7, 4485-661 Vairão, Portugal; 5 UCIBIO—Applied Molecular Biosciences Unit, Toxicologic Pathology Research Laboratory and Associate Laboratory i4HB, Institute for Health and Bioeconomy, University Institute of Health Sciences (1H-TOXRUN, IUCS-CESPU), 4585-116 Gandra, Portugal

**Keywords:** marine microbial diversity, antibiotic resistance, novel bacterial taxa, phylogenetic analysis, environmental resistome, culture-dependent isolation, One Health

## Abstract

Background/Objectives: It is of the utmost importance to study environmental bacteria, as these microorganisms remain poorly characterized regarding their diversity, antimicrobial resistance, and impact on the global ecosystem. This knowledge gap is particularly pronounced for marine bacteria. In this study, we aimed to isolate bacteria from different marine samples and to gain insights into the environmental bacterial resistome, an aspect that remains largely neglected. Methods: Bacteria were isolated from several marine sources using two different culture media, and their identification was based on 16S rRNA gene analysis. Whole-genome sequencing was performed for selected isolates belonging to novel taxa. Antimicrobial susceptibility to seven antibiotics was evaluated using the disk diffusion method. Results: A total of 171 bacterial isolates belonging to the phyla *Pseudomonadota*, *Bacteroidota*, *Planctomycetota*, *Actinomycetota*, and *Bacillota* were obtained from diverse marine samples. The most abundant group belonged to the class *Alphaproteobacteria*. Thirty isolates represented novel taxa, comprising 16 new species and one new genus. Despite the challenges associated with determining antibiotic resistance profiles in environmental bacteria, only one isolate (1.8%) was pan-susceptible, whereas 54 (98.2%) showed resistance to at least one of the tested antibiotics. Moreover, 33 isolates exhibited a multidrug-resistant phenotype. Genome analysis of four novel taxa revealed the presence of an incomplete AdeFGH efflux pump. Conclusions: This study highlights the high bacterial diversity in marine environments, the striking prevalence of antibiotic resistance, and the major methodological challenges in studying environmental bacteria. Importantly, it emphasizes the relevance of culturomics-based approaches for uncovering hidden microbial diversity and characterizing environmental resistomes.

## 1. Introduction

Due to their immense vastness and inaccessibility, the oceans remain significantly less explored in terms of microbial diversity than terrestrial ecosystems [1]. However, since the latter half of the 20th century, advances in molecular and genomic technologies have enabled increasingly detailed investigations into marine microbial communities. The molecular revolution, driven by high-throughput sequencing and metagenomic approaches, has uncovered an immense and largely uncharacterized diversity of oceanic microorganisms [2]. Over the past two decades, thousands of microbial genomes and metagenome-assembled genomes (MAGs) have been generated from marine samples, offering new insights into the metabolic capabilities and ecological roles of marine microorganisms. Despite this progress, many fundamental questions about marine microbial life remain poorly understood [3]. Moreover, alongside metagenomic approaches, the cultivation and isolation of marine microorganisms continue to play a crucial role in deepening our understanding of microbial physiology, ecology, and adaptation, particularly in extreme or polluted environments [4,5]. In fact, culturomics-based approaches have become essential for exploring microbial diversity that remains inaccessible through standard cultivation methods. By enabling the isolation and characterization of previously uncultivable taxa, these methods provide unique insights into microbial ecology and environmental resistomes, highlighting their growing relevance as tools for microbiome research [5].

Coastal marine ecosystems, which serve as dynamic interfaces between land and ocean, are frequently exposed to intense anthropogenic pressures. These include urban development (e.g., destruction of natural habitats), tourism (e.g., increased beach visitation), industrial activities (e.g., fishing and shipping), and, notably, the discharge of untreated or partially treated wastewater [6]. Such pressures can profoundly modulate microbial community composition. Elevated nutrient loads may promote harmful algal blooms (HABs) [7], while increased organic matter can favor the proliferation of heterotrophic bacteria [8]. A particularly pressing concern is the introduction of antibiotics into marine environments, primarily through wastewater discharge and aquaculture effluents [9]. These contaminants not only disrupt native microbial communities but also promote the selection of antibiotic-resistant strains [10]. The combined effects of antibiotic exposure and horizontal gene transfer accelerate the spread of resistance genes, posing significant risks to marine ecosystem health and human populations that depend on these environments [11].

Antibiotic resistance is a complex, interconnected global health challenge, as resistant bacteria and their genes circulate among humans, animals, plants, and the environment without regard for geographical or ecological boundaries [12,13]. This seamless exchange underscores the need for a One Health approach, which recognizes the interdependence of all sectors and calls for integrated surveillance and mitigation strategies [14,15]. Within this framework, marine ecosystems are particularly relevant, as the ocean may act both as a sink and a source of antimicrobial resistance determinants [16]. Understanding the resistome of marine-origin bacteria is, therefore, critical not only for anticipating emerging threats but also for supporting global surveillance efforts aligned with the One Health approach. Importantly, many marine bacteria, although not pathogenic themselves, may harbor mobilizable resistance genes that can be horizontally transferred to clinically relevant organisms, especially in environments where human activities facilitate gene flow [17].

In this study, we isolated bacteria from multiple marine sources collected at a beach in Northern Portugal, characterized their diversity, evaluated their antibiotic resistance profiles, and obtained insights into the environmental resistome.

## 2. Results and Discussion

### 2.1. Bacterial Isolation from Marine Samples

A total of 171 axenic bacterial isolates were obtained from different marine sources collected along the Northern Portuguese coast (Praia da Memória, Matosinhos) (Table S1). Most isolates were recovered from the M607 + NAG medium (*n* = 122; 71.35%), while a smaller number was obtained from the M600 medium (*n* = 49; 28.65%). N-acetylglucosamine (NAG), a structural component of bacterial cell walls, also functions as a signaling molecule and serves as a carbon and nitrogen source, thereby promoting bacterial growth [18].

**Figure 1:**
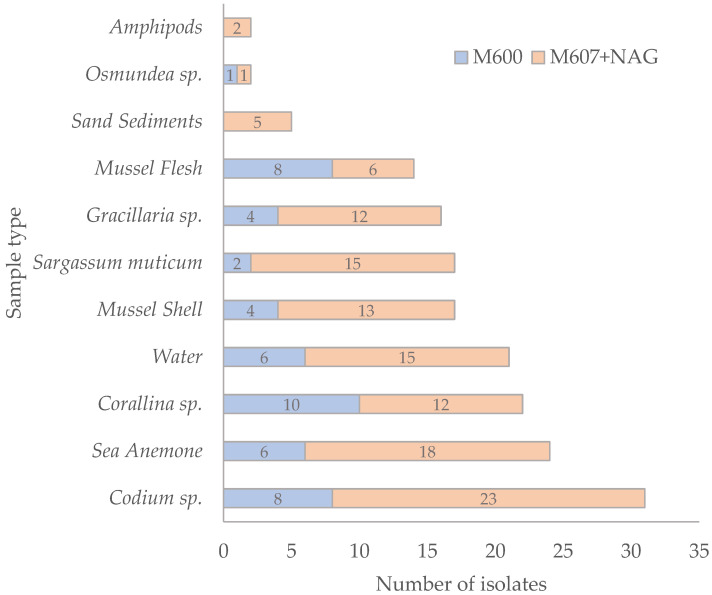
Distribution of bacterial isolates (*n* = 171) obtained from different sampling sources across two culture media (M600 and M607 + NAG). Bars represent the number of isolates per source, separated by the isolation medium used.

To selectively isolate bacteria, the culture media were supplemented with antibiotics. The highest number of isolates was obtained from vancomycin-supplemented media (*n* = 79; 46.2%), followed by imipenem (*n* = 58; 33.9%) and ciprofloxacin (*n* = 34; 19.9%). In terms of sample origin (Figure 1), the top three sources of bacterial isolates were (i) the macroalga *Codium* sp. (*n* = 31; 18.1%), (ii) the sea anemone (*n* = 24; 14.0%), and (iii) the macroalga *Corallina* sp. (*n* = 22; 12.9%). Fewer isolates were recovered from the macroalga *Osmundea* sp. (*n* = 2), amphipods (*n* = 2), and sand sediments (*n* = 5).

### 2.2. Taxonomic Affiliation of the Isolates

**Figure 2:**
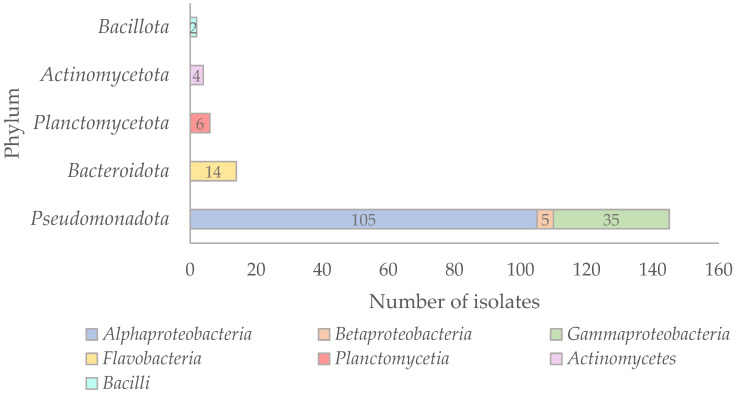
Taxonomic distribution of the 171 bacterial isolates based on 16S rRNA gene sequencing.

The taxonomic affiliation of the 171 isolates was determined by 16S rRNA gene sequencing, followed by BLAST searches against the NCBI database to identify their closest relatives (Table S1). Phylogenetic analyses based on 16S rRNA gene sequences were performed to support the taxonomic assignment of the isolates, and the resulting phylogenetic trees are shown in Figure S1. Regarding the phylogenetic distribution of the isolates, the majority were affiliated with the phylum *Pseudomonadota* (*n* = 145), followed by *Bacteroidota* (*n* = 14), *Planctomycetota* (*n* = 6), *Actinomycetota* (*n* = 4), and *Bacillota* (*n* = 2) (Figure 2).

The two isolates affiliated with the phylum *Bacillota*, PMO135_4.b and PMO127_5, were identified as *Ornithinibacillus contaminans* and *Oceanobacillus profundus*, respectively, with 100% 16S rRNA gene similarity. *Ornithinibacillus contaminans* was originally isolated from human blood [19], while *O. profundus* was isolated from the surface of a deep-sea sediment core and from microbial communities associated with the sponge *Plakortis dariae* [20,21].

Within the phylum *Actinomycetota*, isolates PMO87_15.4.1 and PMO87_15.5 were closely affiliated with “*Mycobacterium adipatum*”, recently described in 2023 [21]. The type strain of “*M. adipatum*” was isolated from petroleum-contaminated soil and is known to possess polycyclic aromatic hydrocarbon (PAH) degradation genes and to degrade phthalates [22,23]. These two isolates also showed high identity (99.37%; Table S1) with *Mycobacterium frederiksbergense*, a species isolated from coal tar-contaminated soil and also capable of degrading PAHs [24]. The other two obtained isolates, PMO133_7 and PMO131_2.1, were affiliated with the species *Microbacterium oxydans*, which has the ability to degrade alginate and laminarin [25], and with *Microbacterium algeriense,* which was isolated from oil field production waters [26]. The genus *Microbacterium* has been reported from a wide range of habitats, including oil-contaminated water, soil, plants, dairy products, insects, steep liquors, human clinical samples, deep-sea sediments, and even from the screen of a mobile phone [26].

Within the phylum *Planctomycetota*, three different species were identified among the six obtained isolates. Isolate PMO112_11.1 was affiliated with *Rubinisphaera brasiliensis*, PMO137_3 with *Gimesia benthica*, and isolates PMO137_10, PMO137_2, PMO137_6, and PMO137_9 with *Novipirellula rosea*. The six isolates showed very high 16S rRNA gene similarity to their closest relatives (99.77–100%; Table S1) and are commonly found in marine environments [27].

All isolates affiliated with the phylum *Bacteroidota* belong to the family *Flavobacteriaceae*. Seven strains were identified as members of the genus *Aquimarina*, mainly related to *A. algiphila*; four isolates were affiliated with the genus *Cellulophaga*, one was affiliated with *Flagellimonas* (formerly *Muricauda*), one was affiliated with *Maribacter*, and one was affiliated with *Zobellia* (Table S1). All these genera are typically associated with marine environments.

**Figure 3:**
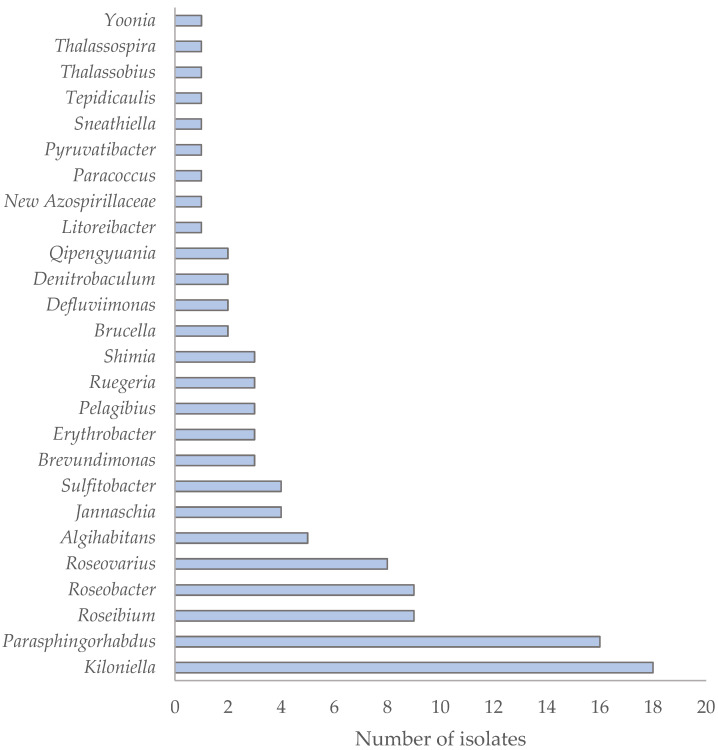
Distribution of 26 genera within the class *Alphaproteobacteria* isolated from various marine environmental sources, based on 105 bacterial isolates.

Within the phylum *Pseudomonadota*, most isolates belonged to the class *Alphaproteobacteria* (*n* = 105; 72.4%), followed by *Gammaproteobacteria* (*n* = 35; 24.1%) and *Βetaproteobacteria* (*n* = 5; 3.5%) (Figure 2). In the first major study of the Sargasso Sea, *Alphaproteobacteria* were found to be the most abundant prokaryotic phylotypes [28], and this group is considered one of the most dominant in marine environments [29,30]. Similarly, in the Coastal NW Mediterranean Sea, *Alphaproteobacteria* have been shown to dominate throughout the year [31]. The 105 *Alphaproteobacteria* isolates found in this study were affiliated with 40 different species across 26 genera, indicating high taxonomic diversity (Figure 3). The most frequently isolated genus was *Kiloniella* (*n* = 18), followed by *Parasphingorhabdua* (*n* = 16). Almost all *Kiloniella* strains were isolated from macroalgae, consistent with the recent description of *Kiloniella laminariae* from the marine macroalga *Saccharina latissima* [32]. Most of the isolates were phylogenetically related to species typically found in aquatic marine habitats (Table S1).

**Figure 4:**
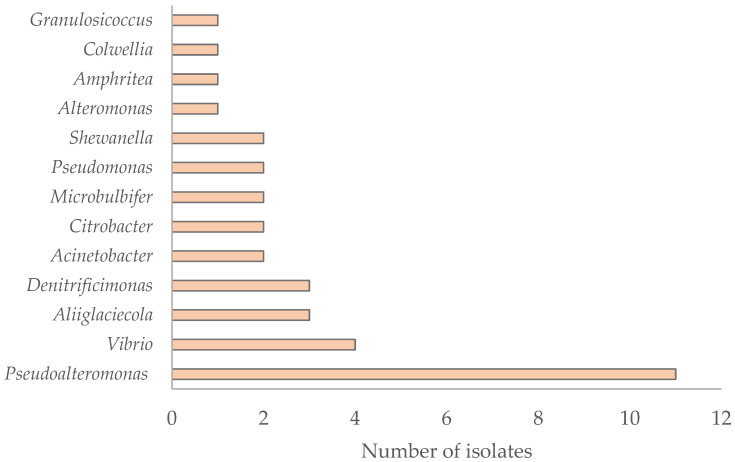
Distribution of 13 genera within the class *Gammaproteobacteria* isolated from various marine environmental sources, based on 35 bacterial isolates.

Among the 13 genera (and 24 species) of the recovered *Gammaproteobacteria*, *Pseudoalteromonas* was the most abundant (*n* = 11) (Figure 4). Most *Gammaproteobacteria* isolates were affiliated with marine-associated species, although some exceptions were observed. Isolates PMO124_1 and PMO94_2 (100% identity) were affiliated with *Acinetobacter johnsonii*, a species known to cause human infections and to harbor antibiotic resistance genes, acting as a potential reservoir of resistance determinants [33,34,35]. Based on sequence analysis, isolate PMO120_1 was closely related to *Citrobacter braakii* (99.92% similarity), whereas isolate PMO126_r.1 was identified as *Citrobacter freundii* with 100% similarity. Both species are members of the *Enterobacteriaceae* family. *C. braakii* has been linked to cases of bacteremia, particularly in patients with comorbidities [36,37,38,39], while *C. freundii* is a commensal of the human gut but can also cause opportunistic infections [40,41]. Three isolates, PMO136_11, PMO107_8.1, and PMO99_9.2, were affiliated with *Vibrio splendidus*, while PMO124_2.1 was affiliated with *Vibrio cyclitrophicus*. Although neither species is currently considered a human pathogen, *V. splendidus* is a well-known fish pathogen [42,43,44].

The five *Betaproteobacteria* isolates showed only distant phylogenetic relationships to *Methylotenera oryzisoli*, with 16S rRNA gene similarity values ranging from 94.92% to 95.56%, indicative of a novel taxon. This species belongs to a recently proposed genus, isolated from rice field soil [45].

### 2.3. Novel Taxa

A total of 30 isolates exhibited 16S rRNA gene sequence identity values below 98.65% relative to their closest known relatives (Table 1), which is the conventional threshold for novel species delimitation [46]. Among these, one isolate showed an identity below 94.5%, which is the commonly accepted cut-off for genus-level delimitation [47] (Table 1). Most of these potential novel taxa were recovered from mussel shells (10 isolates), followed by homogenized sea anemone tissue (8 isolates), and the macroalgae *Codium* sp. (6 isolates). Fewer novel candidates were isolated from *Corallina* sp. (3 isolates) and *Gracilaria* sp. (2 isolates), with one isolate obtained from mussel flesh. Consistent with the overall distribution of isolates, the majority of these potentially novel taxa were retrieved from the M607 + NAG medium (24 isolates), while only six were obtained from the M600 medium. Taxonomically, 2 of the 30 isolates were affiliated with the phylum *Bacteroidota*, while the remaining 28 belonged to the phylum *Pseudomonadota* (19 isolates classified as *Alphaproteobacteria*, 5 as *Βetaproteobacteria*, and 4 as Gammaproteobacteria). In total, these 30 isolates are predicted to represent 16 putative novel species and one potential novel genus, contributing to the discovery of previously undescribed microbial diversity.

### 2.4. Environmental Resistome and One Health Perspective

In this study, phenotypic and genotypic resistance profiles were investigated in 55 marine bacterial isolates selected to ensure taxonomic diversity and representative coverage of the different marine sources and culture conditions sampled. Priority was given to isolates representing distinct phylogenetic lineages, including putative novel taxa, while multiple strains of the same species were also included to assess potential intraspecific variation in resistance behavior.

#### 2.4.1. Challenges in Antimicrobial Resistance Testing of Environmental Isolates

Studying antimicrobial resistance in environmental, non-clinical bacteria presents significant challenges: (i) Aggregative Growth: Many isolates exhibited aggregative behavior, hindering the preparation of uniform bacterial suspensions. Consequently, irregular or spot-like growth was observed on the cultures; (ii) Slow Growth Rates: Most isolates required long incubation times, delaying data observation; (iii) Poor Discernibility: Some isolates formed small, faint colonies, making inhibition zone measurements difficult—even with magnifying tools; (iv) Lack of Standardized Breakpoints: Interpreting inhibition zones is challenging due to the absence of standardized susceptibility breakpoints for non-clinical species; (v) Growth Medium Limitations: The majority of isolates failed to grow on standard Mueller–Hinton II (MH-II) agar. For this reason, susceptibility tests were also performed in M600 medium.

To assess the effect of media composition, reference strains (*Escherichia coli* ATCC 25922 and *Staphylococcus aureus* ATCC 29213) were tested in both MH-II and M600 media. While most results were consistent, some antibiotics (e.g., tetracycline, gentamycin, ciprofloxacin) showed variations in inhibition zones, potentially due to the higher salinity of M600 medium [48] (see Table 1).

#### 2.4.2. Phenotypic Resistance Profiles

Despite all the aforementioned limitations, it was possible to infer interpretable phenotypes for 55 isolates. The criteria from the EUCAST/CLSI guidelines could only be applied to *Acinetobacter* and *Citrobacter* isolates. For the remaining isolates, the absence of an inhibition zone or zones with less than 10 mm was interpreted as resistance. Consequently, most of the 55 environmental isolates tested for antibiotic susceptibility appeared to be resistant to ampicillin, imipenem, tetracycline, and gentamycin, whereas most remained susceptible to cefotaxime and ciprofloxacin (Table 2).

Among the 55 isolates, only one, PMO87_21 (1.8%), was found to be pan-susceptible, while 54 (98.2%) showed resistance to at least one of the antibiotics tested, with 33 isolates exhibiting a multidrug-resistant (MDR) phenotype, defined as resistance to three or more antibiotic classes. In fact, several isolates, including representatives of the genera *Algihabitans*, *Brucella*, *Microbacterium*, *Parashingorhabdus*, and *Stutzerimonas* (Table 2), showed resistance to four or more antibiotics, highlighting their MDR potential. Notably, two isolates, *Zobellia russellii* PMO87_22 and *Microbacterium algeriense* PMO133_7, were resistant to all tested antibiotics.

Isolates affiliated with the phylum *Planctomycetota* also demonstrated high levels of resistance. All four tested strains exhibited resistance to six of the antibiotics used, consistent with previously reported intrinsic resistance patterns in this phylum. For instance, *Novipirellula rosea* strains were only susceptible to ciprofloxacin, while *Rubinisphaera brasiliensis* was only susceptible to tetracycline. These findings align with earlier studies indicating that *Planctomycetota* are naturally resistant to β-lactams (e.g., ampicillin, cefotaxime, imipenem), aminoglycosides (e.g., gentamycin), and glycopeptides (e.g., vancomycin) [49].

When examining species-level trends, variability in resistance patterns was evident, particularly for genera with limited prior information. For example, data on resistance are scarce or absent for several species, including *Algihabitans albus*, *Aquimarina* spp., *Brevundimonas bullata*, *Brevundimonas fontaguae*, and *Erythrobacter rubeus*, which limits direct comparisons.

In the genus *Aliiglaciecola*, the three isolates (PMO87_4.a, PMO87_21, and PMO133_10.1) showed inconsistent behavior against ampicillin, imipenem, and tetracycline (Table 2), diverging from published reports on *A. coringensis*, which has been described as susceptible to ampicillin and tetracycline [50]. Similarly, the susceptibility profiles of the two *Microbulbifer* (PMO140_4 and PMO85_2) species differed, with *M. echini* showing resistance to three antibiotics (ampicillin, imipenem, and tetracycline). Variability in resistance was also observed within the fourteen isolates of *Parashingorhabdus*, especially regarding ampicillin and ciprofloxacin. Nevertheless, all isolates from this genus were uniformly susceptible to cefotaxime and resistant to tetracycline and gentamycin (Table 2). In some cases, the resistance observed can be linked to known genetic determinants. For instance, the type strain of *Parasphingorhabdus cellanae* JHSY0214^T^ harbors multiple resistance genes, including two conferring resistance to fluoroquinolones and seven encoding multidrug efflux pumps [51]. These genomic features may explain the resistance profiles observed in our *Parashingorhabdus* isolates.

Although ciprofloxacin resistance has been reported for some *Brevundimonas* species [52], resistance to carbapenems (e.g., imipenem) observed in *B. fontaguae* (PMO112_3.1) is of concern, as this antibiotic is restricted to the hospital setting and considered a last resort for treatment of severe infections.

Bacteria of the genus *Acinetobacter*, particularly those of the species *Acinetobacter baumannii*, are widely studied and well-known for their high resistance to antibiotics and are considered among the main multidrug-resistant pathogens in hospital environments. Other species, such as *A. johnsonii*, are also opportunistic and can cause infections, especially in hospitalized patients. Curiously, one isolate affiliated with *A. johnsonii* (PMO94_2) was found to be resistant to ciprofloxacin, a fluoroquinolone classified by the World Health Organization (WHO) as a “highest priority critically important antimicrobial”, despite being susceptible to other antibiotics [53].

Similarly, although several studies have demonstrated that *Brucella* species remain in vitro susceptible to most antibiotics and combinations included in the current WHO recommended treatment regimens, such as levofloxacin, tetracyclines, doxycycline, streptomycin, ciprofloxacin, chloramphenicol, gentamicin, tigecycline, and trimethoprim/sulfamethoxazole [54], our results showed that *Brucella rizosphaerae* (PMO87_15.1b) exhibit a clear MDR profile, which is in line with recent findings by Zeb et al. [55].

The two *Citrobacter* isolates (*C. braakii* and *C. freundii*) were resistant to both ampicillin and imipenem, likely due to the presence of chromosomally encoded AmpC β-lactamases and/or horizontally acquired resistance genes, respectively. Additionally, the *C. freundii* isolate exhibited resistance to gentamicin (Table 2).

Although Gram-negative bacteria are intrinsically resistant to vancomycin, several isolates showed in vitro susceptibility (Table 2). This phenomenon, also observed in previous studies [50,56], remains unexplained and warrants further investigation.

Some of the marine bacterial isolates obtained in this study belong to genera such as *Aquimarina*, *Pseudoalteromonas*, and members of the phylum *Planctomycetota*, which are well known for their biosynthetic capabilities, including the production of bioactive compounds such as antibiotics [57,58,59]. This characteristic may contribute to their broad antibiotic resistance profiles.

#### 2.4.3. Genomic Analysis of AMR Genes

In this study, the genomes of four isolates, PMO119_11 (novel genus), PMO90_13 (novel species), PMO138_12 (novel species), and PMO127_3 (novel species), were sequenced and screened for the presence of AMR genes using the CARD-RGI platform. These four isolates represent novel taxa based on 16S rRNA gene divergence and were selected to explore the environmental resistome in under-characterized marine bacteria.

Of the four genomes analyzed, only PMO127_3, an alphaproteobacterium sharing 95.45% 16S rRNA gene similarity with *Tepidicaulis marinus*, lacked any strict hits for AMR genes in the CARD database. In contrast, the genomes of PMO119_11 (92.33% similar to *Inquilinus ginsengisoli*), PMO138_12 (95.32% similar to *Methylotenera oryzisoli*), and PMO90_13 (98.1% similar to *Sneathiella aquamaris*) each carried a strict hit for the *adeF* gene. The *adeF* gene encodes a membrane fusion protein component of the AdeFGH multidrug efflux pump, a resistance mechanism known to confer decreased susceptibility to a broad range of antibiotics, including fluoroquinolones, tetracycline, tigecycline, chloramphenicol, clindamycin, trimethoprim, and sulfamethoxazole [60,61]. Additionally, PMO119_11 carried a strict hit for *fos*A8, a gene encoding a glutathione S-transferase that inactivates fosfomycin, thereby conferring resistance to this antibiotic [62].

Interestingly, despite the presence of the *adeF* gene, no strict hits were detected for the other components of the AdeFGH efflux pump complex in these genomes. Only a loose hit to the *adeG* gene was found in PMO90_13. This incomplete efflux pump, characterized by the presence of *adeF* in the absence of *adeG* and *adeH*, is a pattern previously observed in multiple genomes from non-clinical taxa, including members of the phylum *Planctomycetota* [63].

It is important to note that the CARD-RGI tool is primarily curated for clinically relevant pathogens, which may limit its sensitivity and specificity when applied to environmental or novel bacterial genomes. As such, the absence of strict hits for many isolates may reflect database bias rather than a true lack of resistance genes. Furthermore, the lack of phenotypic susceptibility profiles for these four sequenced strains hinders direct correlation between genotype and phenotype. Nonetheless, these findings suggest that even environmental strains affiliated with putative novel taxa can harbor known resistance determinants, underscoring the ecological relevance of environmental bacteria as potential reservoirs of AMR genes.

## 3. Materials and Methods

### 3.1. Sampling

Marine ecosystems supply a diverse array of goods and services that support human well-being, including food provision and recreational activities [64]. Within coastal areas, antibiotic-resistant pathogenic bacteria can be found in sediments, macroalgae, animals, and seawater [65,66,67,68,69]. Given that several *Planctomycetota* strains have been isolated from sediments, macroalgae, and other beach-associated specimens, our sampling site was Praia da Memória, a beach located in Northern Portugal. Additionally, Praia da Memória is situated near a wastewater treatment plant (WWTP), which may influence the local bacterial diversity and antibiotic-resistance profile.

In March 2020, samples were collected from rocky pools at Praia da Memória (41°13′45.2″ N, 8°43′19.8″ W). Six types of samples were used: seawater, macroalgae, mussels, sandy sediment, a sea anemone, and amphipods.

### 3.2. Media and Culture Conditions

Two different culture media (M607 + NAG and M600; see Table 3) were used to isolate and maintain marine bacteria, initially, with a particular focus on members of the *Planctomycetota* phylum. Medium 607 was supplemented with N-Acetylglucosamine (NAG) (Sigma-Aldrich, St. Louis, MO, USA), as this compound is known to be readily utilized by *Planctomycetota* species. To assess the presence of antibiotic-resistant bacteria in the marine environment, individual culture plates were supplemented with one of the following antibiotics purchased from Sigma-Aldrich (St. Louis, MO, USA): vancomycin (4 µg/mL), imipenem (2 μg/mL), or ciprofloxacin (2 µg/mL). Vancomycin was chosen for two main reasons: (1) its inclusion on the WHO priority list of antibiotic-resistant bacteria, which identifies vancomycin-resistant *Enterococcus faecium* and *S. aureus* as major concerns; and (2) its poor activity against most Gram-negative bacteria due to its limited ability to penetrate the outer membrane. This property makes vancomycin potentially useful for selectively enriching for *Planctomycetota*, which are hypothesized to exhibit intrinsic resistance to several antibiotics. Although current isolation protocols for *Planctomycetota* typically rely on streptomycin and ampicillin [70,71,72,73], no intrinsic resistance mechanisms have been definitively described for any antibiotics in this phylum to date. The vancomycin concentration used in this study was based on the minimum inhibitory concentration breakpoint for resistance in *Enterococcus* spp., as defined by the European Committee on Antimicrobial Susceptibility Testing (EUCAST) [74]. Imipenem, a carbapenem, was included due to its clinical importance and its designation in the WHO priority list, particularly in relation to carbapenem-resistant *A. baumannii*, *Pseudomonas aeruginosa*, and *Enterobacterales*. The concentration used was selected based on previous studies [75]. Ciprofloxacin was also selected based on the WHO priority list, which highlights the emergence of fluoroquinolone resistance in *Salmonella* spp. and *Shigella* spp., both members of the *Enterobacteriaceae* family. The ciprofloxacin concentration was similarly based on values reported in previous studies [76]. To prevent fungal contaminations, all culture media surfaces were sprayed with 1% econazole nitrate (Pevaryl^®^, Karo Pharma AB, Stockholm, Sweden) prior to inoculation.

### 3.3. Sample Processing

Two liters of seawater were collected, and 1 L was filtered through a sterile 0.22 µm pore-size Whatman membrane filter (Whatman, Maidstone, UK). The material retained on the filter was resuspended in 10 mL of sterile seawater, and 100 µL aliquots were inoculated onto the culture media. Additionally, 10 mL of the unfiltered seawater was diluted 1:10 in sterile seawater, and 100 µL of both the undiluted and diluted samples were inoculated onto the culture media, with or without antibiotic supplementation.

Five macroalgae species (*Codium* sp., *Corallina* sp., *Osmundea* sp., *Sargassum* sp., and *Gracilaria* sp.) were collected and processed following previously described protocols with minor modifications [70,71]. To eliminate fungal contaminants, all macroalgal samples were first incubated in 20 mL of sterile seawater containing econazole nitrate powder (Pevaryl^®^) for 1 h under agitation. Samples were then rinsed with sterile natural seawater before being cut into small pieces or having their surfaces scraped. The scraped material was resuspended in 1 mL of sterile seawater, and 100 µL of the suspension, along with algal fragments, were inoculated onto the culture media with or without antibiotic supplementation.

For mussel samples (*Mytilus* sp.), both the shell surface biofilm and the flesh were processed. The biofilm was collected by scraping the shell with a sterile swab, which was then placed in a tube with sterile seawater, vortexed, and incubated under agitation for 1 h. Meanwhile, the mussel flesh was homogenized in sterile seawater with a sterile mortar. From each type of sample, 100 µL aliquots were inoculated onto the culture media with or without antibiotic supplementation.

The sea anemone was washed in sterile natural seawater and homogenized using a sterile mortar. From the resulting homogenate, 100 µL were inoculated onto the culture media with and without antibiotics.

The amphipods were directly placed on the culture media supplemented with or without antibiotics.

Sandy sediment samples were collected in sterile tubes and subjected to an enrichment-based approach. One gram of sand was added to two separate tubes, each containing 25 mL of M600 or M607 + NAG medium supplemented with 4 µg/mL vancomycin and econazole nitrate (Pevaryl^®^). The cultures were incubated at 25 °C with shaking at 220 rpm for several months. After incubation, 100 µL aliquots were plated onto selective culture media, with or without antibiotic supplementation.

All cultures in M600 and M607 + NAG media were incubated at 26 °C in the dark and routinely checked for colony formation. Different colony morphotypes were isolated, and axenic cultures were stored at −80 °C in their respective culture media with 20% (*v*/*v*) glycerol (Sigma-Aldrich, St. Louis, MO, USA).

### 3.4. Bacterial Identification by Molecular Analysis

Genomic DNA from pure bacterial isolates was extracted using the E.Z.N.A.^®^ Bacterial DNA Kit (Omega Bio-Tek, Norcross, GA, USA), according to the manufacturer’s instructions. For each isolate, the extracted DNA was used as a template for PCR amplification of the 16S rRNA gene, a phylogenetic marker, using the universal primers 27F and 1492R [78].

Each 25 µL PCR reaction contained 12.5 μL of NZYTaq 2× Green Master Mix, 0.25 μL of each primer (0.1 µM), 2 μL of DNA template, and 10 μL of nuclease-free water. PCR amplification was carried out in a MyCyclerTM Thermal Cycler (Bio-Rad, Hercules, CA, USA) with the following thermal profile: an initial denaturation at 95 °C for 5 min, followed by 30 cycles of denaturation at 95 °C for 1 min, annealing at 56 °C for 1 min, and extension at 72 °C for 1.5 min, with a final extension step at 72 °C for 10 min. PCR products were visualized by electrophoresis on a 0.8% agarose gel in 1× Tris-acetate-EDTA (TAE) buffer and stained with 0.4 µL of GreenSafe Premium (NZYTech, Lisbon, Portugal) per 100 mL of TAE.

All amplicons were subsequently purified using the GFX PCR DNA and Gel Band Purification Kit (GE Healthcare, Chicago, IL, USA) prior to sequencing, which was performed at GATC Biotech (Konstanz, Germany). The resulting sequences were trimmed and analyzed using Geneious Prime 2021.

To determine phylogenetic affiliation, the obtained 16S rRNA gene sequences were compared against the NCBI Genbank database [79] using the NCBI Standard Nucleotide BLAST tool (version 2.14.0; NCBI), in order to identify species and determine the closest phylogenetic relatives.

Phylogenetic analyses were conducted using MEGA7 software (version 7.0) [80]. 16S rRNA gene sequences of the isolates were compared with those of closely related types retrieved from the LPSN database [81] and GenBank. Sequences were aligned using ClustalW software [82]. Phylogenetic trees were constructed using the Maximum Likelihood method under the General Time Reversible (GTR) model with gamma-distributed rates and invariant sites (G + I) [83]. The robustness of the inferred phylogenies was assessed using 1000 bootstrap replicates.

### 3.5. Genome Sequencing, Assembly, and Analysis

Four bacterial isolates, each representing a different genus based on 16S rRNA gene analysis, were selected for genome sequencing. Isolates PMO119_11, PMO90_13, and PMO138_12 were sequenced using Oxford Nanopore technology, while isolate PMO127_3 was sequenced using the MiSeq system (Illumina, San Diego, CA, USA)). De novo genome assembly of PMO127_3 was performed using CLC Genomics Workbench (QIAGEN), version 21.0.1. In all cases, open reading frames (ORFs) were predicted using Prodigal, version 2.6.3 [84], and the resulting coding sequences were annotated with Prokka, version 1.14.6 [85]. The completeness and contamination of the assembled genomes were assessed using checkM, version 1.20 [86].

The presence of antibiotic resistance genes in the sequenced genomes was evaluated with the CARD-RGI platform, using the genomic DNA sequences as input. Only perfect and strict hits were considered, and the option to nudge ≥ 95% identity loose hits to strict was excluded [39,40,87].

### 3.6. Antibiotic Susceptibility Testing

The antibiotic susceptibility of the isolates was evaluated using the Kirby–Bauer disk diffusion method [88], with *E. coli* ATCC 25922 and *S. aureus* ATCC 29213 included as quality control strains. Tests were performed on standard Mueller-Hinton II medium (Oxoid™, Thermo Fisher Scientific, Basingstoke, UK) using a panel of clinically relevant antibiotics representing different major classes (Table 2). For isolates that were unable to grow on Mueller–Hinton II, a modified Kirby–Bauer method was applied, using their specific isolation media (M607 + NAG or M600), as previously described [49]. Results were validated and, when applicable, interpreted according to the guidelines provided in the 17th informational supplement of the Performance Standards for Antimicrobial Susceptibility Testing [89].

## 4. Conclusions

This study enabled the isolation of a substantial and diverse collection of marine bacteria (*n* = 171) from Praia da Memória, encompassing representatives from five different phyla. Notably, 30 of these isolates corresponded to 16 novel species and one novel genus. These findings underscore the value of exploring underrepresented habitats and reinforce the importance of further polyphasic taxonomic characterization, including morphological, physiological, and genomic analyses, to formally describe and classify these novel organisms.

Our work also highlights the challenges associated with assessing AMR in environmental bacterial isolates, particularly when relying on phenotypic assays. Factors such as atypical growth patterns, slow-growing strains, and the absence of standardized interpretive criteria for environmental species complicate the assessment. Despite these limitations, culture-dependent methods proved essential in capturing bacterial diversity not accessible by culture-independent techniques. Importantly, this approach enabled the isolation of several previously uncultured taxa, allowing for a deeper exploration of their phenotypic characteristics, including antibiotic susceptibility, and contributing novel data to the environmental resistome.

In addition, many of the genera identified in this study have previously been associated with hydrocarbon degradation. Considering the geographic proximity of the sampling site to an oil refinery and its wastewater treatment plant outflow, the recovery of such bacteria is not unexpected. These findings suggest that the bacterial collection assembled here might hold significant biotechnological potential, particularly for applications in bioremediation of hydrocarbon-contaminated environments.

Overall, this study contributes valuable new insights into the diversity, taxonomy, and ecology of marine bacteria, expands our knowledge of their resistance profiles, and lays the groundwork for both novel species descriptions and the investigation of biotechnological applications related to environmental health and One Health approaches.

## Figures and Tables

**Table 1:** Taxonomic characterization and closest phylogenetic relatives of marine bacterial isolates.

Isolate	NCBI Accession Number	Sample Source	Phylum	Class	Closest Taxa	% Identity *
PMO121_14	PP079866	Mussel Shell	*Bacteroidota*	*Flavobacteriia*	*Aquimarina algiphila* strain 9Alg 151	96.16
PMO114_2.a	PP079845	*Codium* sp.	*Bacteroidota*	*Flavobacteriia*	*Muricauda koreensis* strain ECD12	98.61
PMO140_13	PP079889	Mussel Shell	*Pseudomonadota*	*Alphaproteobacteria*	*Defluviimonas aestuarii* strain BS14	97.22
PMO140_20.1.b	PP079880	Mussel Shell	*Pseudomonadota*	*Alphaproteobacteria*	*Defluviimonas aestuarii* strain BS14	97.38
PMO121_15.2	PP079843	Mussel Shell	*Pseudomonadota*	*Alphaproteobacteria*	*Jannaschia seosinensis* strain CL-SP26	98.21
PMO121_15.5	PP079842	Mussel Shell	*Pseudomonadota*	*Alphaproteobacteria*	*Jannaschia seosinensis* strain CL-SP26	98.16
PMO140_2.r	PP079893	Mussel Shell	*Pseudomonadota*	*Alphaproteobacteria*	*Jannaschia seosinensis* strain CL-SP26	97.48
PMO140_6	PP079883	Mussel Shell	*Pseudomonadota*	*Alphaproteobacteria*	*Jannaschia seosinensis* strain CL-SP26	98.21
PMO110_11	PP079825	Sea Anemone	*Pseudomonadota*	*Alphaproteobacteria*	*Litoreibacter meonggei* strain MA1-1	98.46
PMO119_11	OK103952	Sea Anemone	*Pseudomonadota*	*Alphaproteobacteria*	*Inquilinus ginsengisoli* strain Gsoil 080	92.33
PMO102_6.2	PP079808	Mussel Shell	*Pseudomonadota*	*Alphaproteobacteria*	*Parasphingorhabdus litoris* DSM 22379	98.4
PMO123_3.2	PP079787	*Codium* sp.	*Pseudomonadota*	*Alphaproteobacteria*	*Pyruvatibacter mobilis* CGMCC 1.15125	98
PMO121_3.1	PP079785	Mussel Shell	*Pseudomonadota*	*Alphaproteobacteria*	*Qipengyuania seohaensis* strain SW-135	98.57
PMO114_18	PP079776	*Codium* sp.	*Pseudomonadota*	*Alphaproteobacteria*	*Roseobacter cerasinus* strain AI77	98.64
PMO114_5.2	PP079773	*Codium* sp.	*Pseudomonadota*	*Alphaproteobacteria*	*Roseobacter cerasinus* strain AI77	98.31
PMO132_8.1	PP079774	*Codium* sp.	*Pseudomonadota*	*Alphaproteobacteria*	*Roseobacter cerasinus* strain AI77	98.34
PMO138_13.1	PP079882	Sea Anemone	*Pseudomonadota*	*Alphaproteobacteria*	*Shimia thalassica* strain CECT 7735	97.49
PMO90_13	OK103951	*Gracilaria* sp.	*Pseudomonadota*	*Alphaproteobacteria*	*Sneathiella aquamaris* 216LB-ZA1-12	98.1
PMO140_12	PP079890	Mussel Shell	*Pseudomonadota*	*Alphaproteobacteria*	*Sulfitobacter marinus* strain SW-265	98.14
PMO127_3	OK103955	*Gracilaria* sp.	*Pseudomonadota*	*Alphaproteobacteria*	*Tepidicaulis marinus* strain MA2	95.45
PMO138_12	OK103953	Sea Anemone	*Pseudomonadota*	*Betaproteobacteria*	*Methylotenera oryzisoli* strain La3113	95.32
PMO138_15.2	PP079900	Sea Anemone	*Pseudomonadota*	*Betaproteobacteria*	*Methylotenera oryzisoli* strain La3113	95.49
PMO138_17	PP079899	Sea Anemone	*Pseudomonadota*	*Betaproteobacteria*	*Methylotenera oryzisoli* strain La3113	95.33
PMO138_18	PP079898	Sea Anemone	*Pseudomonadota*	*Betaproteobacteria*	*Methylotenera oryzisoli* strain La3113	94.92
PMO138_2	PP079903	Sea Anemone	*Pseudomonadota*	*Betaproteobacteria*	*Methylotenera oryzisoli* strain La3113	95.56
PMO133_10.1	PP079870	*Corallina* sp.	*Pseudomonadota*	*Gammaproteobacteria*	*Aliiglaciecola litoralis* strain Sd 2-38	95.95
PMO87_21	PP079871	*Corallina* sp.	*Pseudomonadota*	*Gammaproteobacteria*	*Aliiglaciecola litoralis* strain Sd 2-38	96.19
PMO87_4.a	PP079872	*Corallina* sp.	*Pseudomonadota*	*Gammaproteobacteria*	*Aliiglaciecola litoralis* strain Sd 2-38	95.6
PMO114_2.T	PP079869	*Codium* sp.	*Pseudomonadota*	*Gammaproteobacteria*	*Alteromonas alba* strain 190	94.61
PMO85_2	PP079870	*Corallina* sp.	*Pseudomonadota*	*Gammaproteobacteria*	*Microbulbifer echini* strain AM134	97.94

* Taxonomic identification was based on 16S rRNA gene sequence similarity to reference sequences deposited in the NCBI database. Values < 98.65% indicate distinct species, while values < 94.5% indicate distinct genera, as observed for isolate PMO119_11.

**Table 2:** Antimicrobial susceptibility profiles and taxonomic affiliation of 55 bacterial isolates from different marine sources.

Isolate	Sample Source	Closest Species	% Identity *	Phylum/Class	Inhibition Zone Diameter (mm)						
					AMP	CTX	IPM	VAN	TET	GEN	CIP
PMO87_15.4.1	*Corallina* sp.	*Mycobacterium adipatum*	99.37	*Actinomycetota/Actinomycetia*	31	NM	40	10	0	0	42
PMO133_7	*Corallina* sp.	*Microbacterium algeriense*	99.89	*Actinomycetota/Actinomycetia*	0	0	0	19	0	0	0
PMO90_19.1	*Gracilaria* sp.	*Aquimarina algiphila*	99.15	*Bacteroidota/Flavobacteriia*	ND	ND	ND	21	16	10	49
132_20.2.3	*Codium* sp.	*Aquimarina macrocephali*	100	*Bacteroidota/Flavobacteriia*	10	22	0	20	0	ND	66
PMO87_22	*Corallina* sp.	*Zobellia russellii*	99.92	*Bacteroidota/Flavobacteriia*	8	0	0	11	8	0	9
PMO137_2	Sediments	*Novipirellula rosea*	99.77	*Planctomycetota/Planctomycetia*	0	0	0	0	0	0	34
PMO137_6	Sediments	*Novipirellula rosea*	99.77	*Planctomycetota/Planctomycetia*	0	0	0	0	0	0	40
PMO137_9	Sediments	*Novipirellula rosea*	99.78	*Planctomycetota/Planctomycetia*	0	0	0	0	0	0	32
PMO112_11.1.l	Mussel Shell	*Rubinisphaera brasiliensis*	100	*Planctomycetota/Planctomycetia*	0	0	0	0	32	0	0
PMO100_1.1	Sea Anemone	*Algihabitans albus*	100	*Pseudomonadota/Alphaproteobacteria*	17	0	0	0	11	0	27
PMO94_4	Seawater	*Brevundimonas bullata*	99.86	*Pseudomonadota/Alphaproteobacteria*	10	21	24	0	8	20	0
PMO122_3.1	Seawater	*Brevundimonas fontaquae*	99.66	*Pseudomonadota/Alphaproteobacteria*	13	30	0	ND	9	0	0
PMO87_15.1.b	*Corallina* sp.	*Brucella rhizosphaerae*	99.68	*Pseudomonadota/Alphaproteobacteria*	0	26	0	0	0	0	17
PMO140_13	Mussel Shell	*Defluviimonas aestuarii*	97.22	*Pseudomonadota/Alphaproteobacteria*	0	22	14	0	18	12	66
PMO112_8.2	Mussel Shell	*Erythrobacter rubeus*	98.97	*Pseudomonadota/Alphaproteobacteria*	NM	NM	0	27	0	0	40
PMO140_15.2	Mussel Shell	*Erythrobacter rubeus*	98.99	*Pseudomonadota/Alphaproteobacteria*	48	60	0	27	8	0	43
PMO139_7.1	Mussel Interior	*Erythrobacter rubeus*	99.03	*Pseudomonadota/Alphaproteobacteria*	NM	NM	NM	21	0	0	44
PMO121_15.5	Mussel Shell	*Jannaschia seosinensis*	98.16	*Pseudomonadota/Alphaproteobacteria*	NM	NM	14	15	7	15	NM
PMO140_2.r	Mussel Shell	*Jannaschia seosinensis*	97.48	*Pseudomonadota/Alphaproteobacteria*	NM	NM	NM	16	7	20	NM
PMO102_1.1	Mussel Shell	*Parasphingorhabdus cellanae*	99.92	*Pseudomonadota/Alphaproteobacteria*	0	34	0	26	0	0	0
PMO95_5.L.1	*Codium* sp.	*Parasphingorhabdus litoris*	99.6	*Pseudomonadota/Alphaproteobacteria*	0	31	0	22	0	0	0
PMO114_1.a	*Codium* sp.	*Parasphingorhabdus cellanae*	99.85	*Pseudomonadota/Alphaproteobacteria*	0	44	0	12	0	0	20
PMO114_13	*Codium* sp.	*Parasphingorhabdus cellanae*	99.92	*Pseudomonadota/Alphaproteobacteria*	0	54	0	27	0	0	30
PMO114_16.1	*Codium* sp.	*Parasphingorhabdus cellanae*	100	*Pseudomonadota/Alphaproteobacteria*	0	44	0	13	0	0	23
PMO115_16.1	*Corallina* sp.	*Parasphingorhabdus cellanae*	99.05	*Pseudomonadota/Alphaproteobacteria*	0	35	0	29	0	0	16
PMO96_5.1	*Corallina* sp.	*Parasphingorhabdus cellanae*	100	*Pseudomonadota/Alphaproteobacteria*	NM	NM	NM	36	0	0	28
PMO95_8.1	*Codium* sp.	*Parasphingorhabdus cellanae*	99.92	*Pseudomonadota/Alphaproteobacteria*	28	NM	NM	25	0	8	19
PMO95_13.2	*Codium* sp.	*Parasphingorhabdus cellanae*	99.84	*Pseudomonadota/Alphaproteobacteria*	10	22	30	32	8	0	15
PMO123_1	*Codium* sp.	*Parasphingorhabdus cellanae*	99.19	*Pseudomonadota/Alphaproteobacteria*	0	47	0	27	0	0	0
PMO123_2	*Codium* sp.	*Parasphingorhabdus cellanae*	99.07	*Pseudomonadota/Alphaproteobacteria*	0	50	0	22	0	0	0
PMO95_11	*Codium* sp.	*Parasphingorhabdus cellanae*	99.69	*Pseudomonadota/Alphaproteobacteria*	20	NM	NM	26	0	0	0
PMO95_10	*Codium* sp.	*Parasphingorhabdus cellanae*	100	*Pseudomonadota/Alphaproteobacteria*	24	NM	NM	26	7	0	28
PMO140_9.1.2	Mussel Shell	*Parasphingorhabdus cellanae*	99.92	*Pseudomonadota/Alphaproteobacteria*	0	42	0	27	0	0	0
PMO114_8.v	*Codium* sp.	*Qipengyuania gelatinilytica*	98.75	*Pseudomonadota/Alphaproteobacteria*	46	56	0	32	9	11	43
PMO110_1.1	Sea Anemone	*Roseovarius aestuarii*	99.28	*Pseudomonadota/Alphaproteobacteria*	0	40	14	0	9	0	48
PMO111_2.1	Mussel Interior	*Ruegeria faecimaris*	98.96	*Pseudomonadota/Alphaproteobacteria*	39	52	17	11	8	0	46
PMO90_13	*Gracilaria* sp.	*Sneathiella aquimaris*	98.1	*Pseudomonadota/Alphaproteobacteria*	0	32	46	0	9	11	12
PMO122_2.a	Seawater	*Yoonia maritima*	98.78	*Pseudomonadota/Alphaproteobacteria*	NM	NM	NM	18	10	26	48
PMO94_2	Seawater	*Acinetobacter johnsonii*	100	*Pseudomonadota/Gammaproteobacteria*	22	23	40	ND	20	27	0
PMO87_4.a	*Corallina* sp.	*Aliiglaciecola litoralis*	95.6	*Pseudomonadota/Gammaproteobacteria*	16	27	25	18	8	13	25
PMO87_21	*Corallina* sp.	*Aliiglaciecola litoralis*	96.19	*Pseudomonadota/Gammaproteobacteria*	13	41	26	18	21	20	32
PMO133_10.1	*Corallina* sp.	*Aliiglaciecola litoralis*	95.95	*Pseudomonadota/Gammaproteobacteria*	0	40	0	21	8	15	31
PMO120_1	Mussel Interior	*Citrobacter braakii*	99.92	*Pseudomonadota/Gammaproteobacteria*	6	32	15	ND	18	17	40
PMO126_r.1	*Sargassum muticum*	*Citrobacter freundii*	100	*Pseudomonadota/Gammaproteobacteria*	10	29	15	ND	18	15	20
PMO140_4	Mussel Shell	*Microbulbifer echini*	100	*Pseudomonadota/Gammaproteobacteria*	0	47	0	12	0	15	35
PMO85_2	Seawater	*Microbulbifer okhotskensis*	99.05	*Pseudomonadota/Gammaproteobacteria*	0	21	33	0	20	18	40
PMO113_2	Seawater	*Pseudoalteromonas prydzensis*	99.06	*Pseudomonadota/Gammaproteobacteria*	14	24	0	0	0	10	23
PMO141_1.v	Invertebrate	*Pseudoalteromonas translucida*	100	*Pseudomonadota/Gammaproteobacteria*	17	0	0	12	9	10	31
PMO88_1	*Osmundea* sp.	*Pseudoalteromonas arctica*	99.78	*Pseudomonadota/Gammaproteobacteria*	20	25	25	0	13	11	24
PMO87_5.2	*Corallina* sp.	*Pseudoalteromonas translucida*	100	*Pseudomonadota/Gammaproteobacteria*	18	25	21	0	12	10	24
PMO85_3.1	Seawater	*Pseudoalteromonas carrageenovora*	100	*Pseudomonadota/Gammaproteobacteria*	21	37	29	10	13	11	35
PMO103_8	Seawater	*Denitrificimonas caeni*	99.61	*Pseudomonadota/Gammaproteobacteria*	19	0	34	0	0	20	26
PMO94_5	Seawater	*Denitrificimonas caeni*	99.35	*Pseudomonadota/Gammaproteobacteria*	ND	ND	ND	0	0	17	0
PMO108_1.1.l	*Gracillaria* sp.	*Shewanella colwelliana*	100	*Pseudomonadota/Gammaproteobacteria*	ND	ND	0	0	0	10	32
PMO138_9.1	Sea Anemone	*Stutzerimonas nitrititolerans*	99.75	*Pseudomonadota/Gammaproteobacteria*	0	18	0	0	0	20	22

* Percentage of 16S rRNA gene sequence identity to the closest related species. AMP, ampicillin; CTX, cefotaxime; IPM, imipenem; VAN, vancomycin; TET, tetracycline; GEN, gentamicin; CIP, ciprofloxacin. ND, not determined; NM, inhibition zone not measurable due to excessive size. Resistance profiles are highlighted in red according to the criterion of an inhibition zone ≤ 10 mm; specifically, for *Acinetobacter* and *Citrobacter* species, resistance was interpreted according to CLSI guidelines.

**Table 3:** Composition of the culture media (M600 and M607 + NAG) used for bacterial isolation.

Component	M600 (per 1000 mL)	M607 + NAG (per 1000 mL)
Peptone	1 g	0.25 g
Yeast Extract	1 g	0.25 g
Tris-HCl	50 mL	50 mL
Glucose (2.5%)	40 mL	10 mL
Hutner’s Basal Salts *	20 mL	20 mL
Vitamin Mix **	10 mL	10 mL
NAG (5%)	–	10 mL
Agar	16 g	16 g
Natural Seawater	880 mL	900 mL

* Hutner’s Basal Salts according to Cohen-Bazire et al. [77]. ** Vitamin Mix contains 0.1 µg/mL cyanocobalamin, 2.0 µg/mL biotin, 5.0 µg/mL thiamine-HCl, 5.0 µg/mL Ca-pantothenate, 2.0 µg/mL folic acid, 5.0 µg/mL riboflavin, and 5.0 µg/mL nicotinamide.

## Data Availability

Data are contained within the article and Supplementary Materials.

## Supplementary Materials

The following supporting information can be downloaded at https://www.mdpi.com/article/10.3390/antibiotics15010110/s1. Figure S1: Phylogenetic trees; Table S1: Phylogenetic characterization of the 171 isolates.
