# Supplementary material for: Exploring the Environmental Resistome and Bacterial Novelty in Marine Isolates from the North Portuguese Coast
Source: Antibiotics (Basel). 2026 Jan 22;15(1):110. doi: 10.3390/antibiotics15010110 (PMC12838003; doi:10.3390/antibiotics15010110)
Supplement: Supplementary file 1 [file antibiotics-15-00110-s001.zip › FigureS1_.pdf]

a)

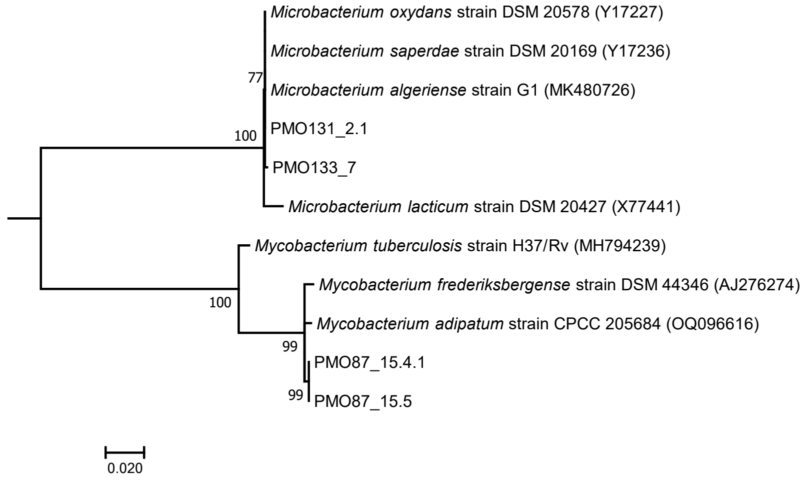
Microbacterium oxydans strain DSM 20578 (Y17227)
Microbacterium saperdae strain DSM 20169 (Y17236)
Microbacterium algeriense strain G1 (MK480726)
PMO131_2.1
PMO133_7
Microbacterium lacticum strain DSM 20427 (X77441)
Mycobacterium tuberculosis strain H37/Rv (MH794239)
Mycobacterium frederiksbergense strain DSM 44346 (AJ276274)
Mycobacterium adipatum strain CPCC 205684 (OQ096616)
PMO87_15.4.1
PMO87_15.5
77
100
100
99
99
0.020

b)

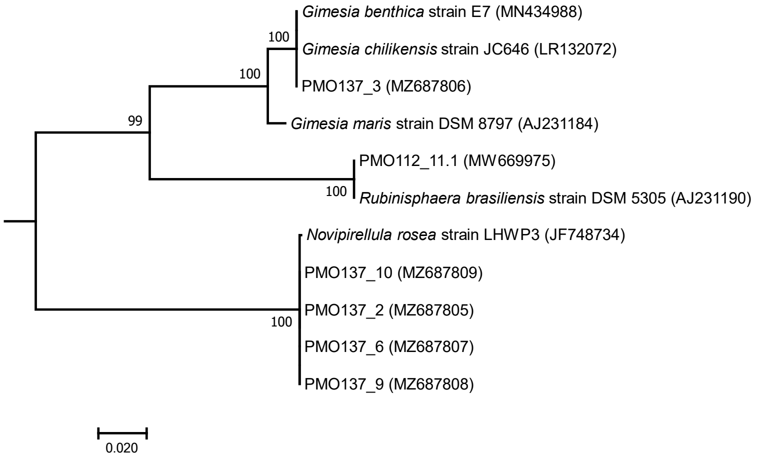
Gimesia benthica strain E7 (MN434988)
Gimesia chilikensis strain JC646 (LR132072)
PMO137_3 (MZ687806)
Gimesia maris strain DSM 8797 (AJ231184)
PMO112_11.1 (MW669975)
Rubinisphaera brasiliensis strain DSM 5305 (AJ231190)
Novipirellula rosea strain LHWP3 (JF748734)
PMO137_10 (MZ687809)
PMO137_2 (MZ687805)
PMO137_6 (MZ687807)
PMO137_9 (MZ687808)
100
100
99
100
100
0.020

c)

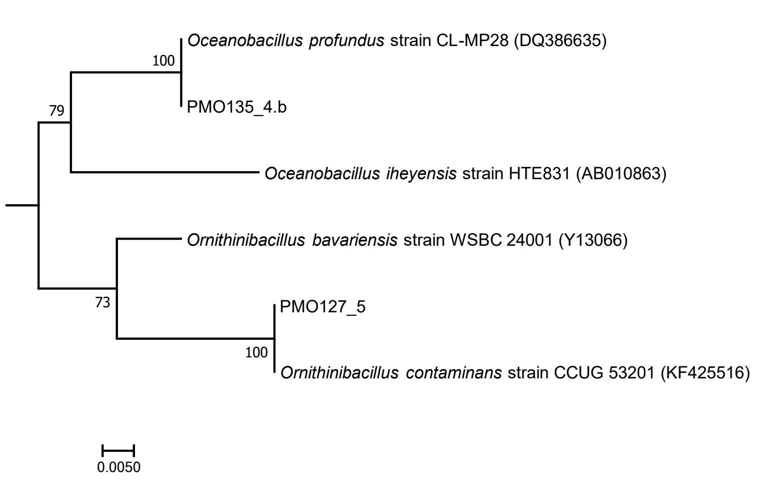
Oceanobacillus profundus strain CL-MP28 (DQ386635)
PMO135_4.b
Oceanobacillus iheyensis strain HTE831 (AB010863)
Ornithinibacillus bavariensis strain WSBC 24001 (Y13066)
PMO127_5
Ornithinibacillus contaminans strain CCUG 53201 (KF425516)
100
79
73
100
0.0050

d)

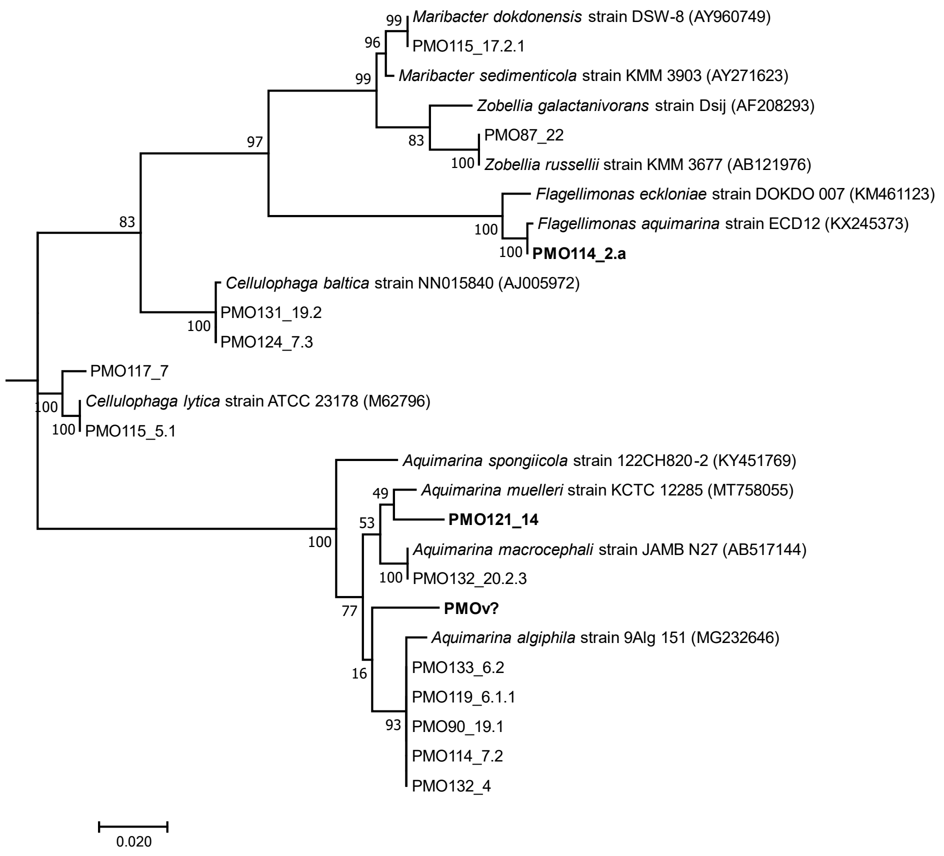

Maribacter dokdonensis strain DSW-8 (AY960749)
PMO115_17.2.1
Maribacter sedimenticola strain KMM 3903 (AY271623)
Zobellia galactanivorans strain Dsij (AF208293)
PMO87_22
Zobellia russellii strain KMM 3677 (AB121976)
Flagellimonas eckloniae strain DOKDO 007 (KM461123)
Flagellimonas aquimarina strain ECD12 (KX245373)
PMO114_2.a
Cellulophaga baltica strain NN015840 (AJ005972)
PMO131_19.2
PMO124_7.3
PMO117_7
Cellulophaga lytica strain ATCC 23178 (M62796)
PMO115_5.1
Aquimarina spongiicola strain 122CH820-2 (KY451769)
Aquimarina muelleri strain KCTC 12285 (MT758055)
PMO121_14
Aquimarina macrocephali strain JAMB N27 (AB517144)
PMO132_20.2.3
PMOv?
Aquimarina algiphila strain 9Alg 151 (MG232646)
PMO133_6.2
PMO119_6.1.1
PMO90_19.1
PMO114_7.2
PMO132_4
99
96
99
97
83
83
100
100
100
100
100
100
100
49
53
100
100
77
16
93
0.020

e)

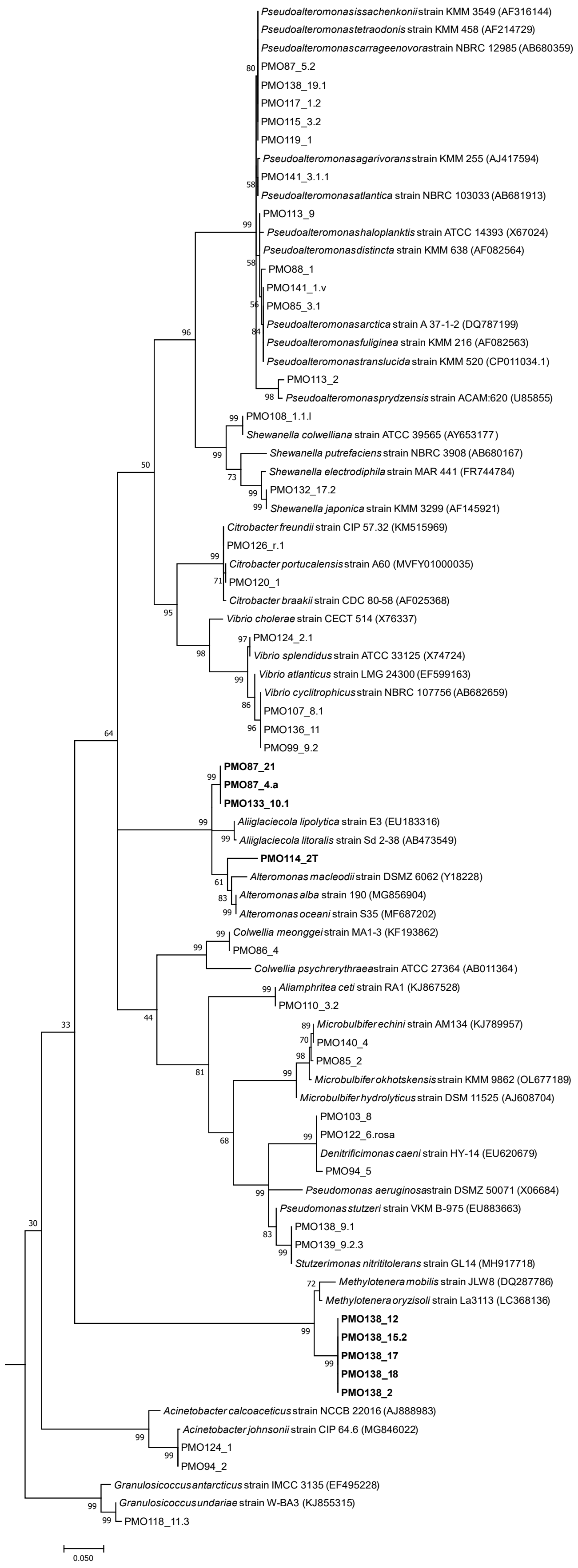

Pseudoalteromonas issachenkonii strain KMM 3549 (AF316144)
Pseudoalteromonas tetraodonis strain KMM 458 (AF214729)
Pseudoalteromonas carrageenovora strain NBRC 12985 (AB680359)
PMO87_5.2
PMO138_19.1
PMO117_1.2
PMO115_3.2
PMO119_1
Pseudoalteromonas agarivorans strain KMM 255 (AJ417594)
PMO141_3.1.1
Pseudoalteromonas atlantica strain NBRC 103033 (AB681913)
PMO113_9
Pseudoalteromonas haloplanktis strain ATCC 14393 (X67024)
Pseudoalteromonas distincta strain KMM 638 (AF082564)
PMO88_1
PMO141_1.v
PMO85_3.1
Pseudoalteromonas arctica strain A 37-1-2 (DQ787199)
Pseudoalteromonas fuliginea strain KMM 216 (AF082563)
Pseudoalteromonas translucida strain KMM 520 (CP011034.1)
PMO113_2
Pseudoalteromonas prydzensis strain ACAM:620 (U85855)
PMO108_1.1.l
Shewanella colwelliana strain ATCC 39565 (AY653177)
Shewanella putrefaciens strain NBRC 3908 (AB680167)
Shewanella electrodiphila strain MAR 441 (FR744784)
PMO132_17.2
Shewanella japonica strain KMM 3299 (AF145921)
Citrobacter freundii strain CIP 57.32 (KM515969)
PMO126_r.1
Citrobacter portucalensis strain A60 (MVFY01000035)
PMO120_1
Citrobacter braakii strain CDC 80-58 (AF025368)
Vibrio cholerae strain CECT 514 (X76337)
PMO124_2.1
Vibrio splendidus strain ATCC 33125 (X74724)
Vibrio atlanticus strain LMG 24300 (EF599163)
Vibrio cyclitrophicus strain NBRC 107756 (AB682659)
PMO107_8.1
PMO136_11
PMO99_9.2
PMO87_21
PMO87_4.a
PMO133_10.1
Aliiglaciecola lipolytica strain E3 (EU183316)
Aliiglaciecola litoralis strain Sd 2-38 (AB473549)
PMO114_2T
Alteromonas macleodii strain DSMZ 6062 (Y18228)
Alteromonas alba strain 190 (MG856904)
Alteromonas oceani strain S35 (MF687202)
Colwellia meonggei strain MA1-3 (KF193862)
PMO86_4
Colwellia psychrerythraea strain ATCC 27364 (AB011364)
Aliamphritea ceti strain RA1 (KJ867528)
PMO110_3.2
Microbulbifer echini strain AM134 (KJ789957)
PMO140_4
PMO85_2
Microbulbifer okhotskensis strain KMM 9862 (OL677189)
Microbulbifer hydrolyticus strain DSM 11525 (AJ608704)
PMO103_8
PMO122_6.rosa
Denitrificimonas caeni strain HY-14 (EU620679)
PMO94_5
Pseudomonas aeruginosa strain DSMZ 50071 (X06684)
Pseudomonas stutzeri strain VKM B-975 (EU883663)
PMO138_9.1
PMO139_9.2.3
Stutzerimonas nitrititolerans strain GL14 (MH917718)
Methylotenera mobilis strain JLW8 (DQ287786)
Methylotenera oryzisoli strain La3113 (LC368136)
PMO138_12
PMO138_15.2
PMO138_17
PMO138_18
PMO138_2
Acinetobacter calcoaceticus strain NCCB 22016 (AJ888983)
Acinetobacter johnsonii strain CIP 64.6 (MG846022)
PMO124_1
PMO94_2
Granulosicoccus antarcticus strain IMCC 3135 (EF495228)
Granulosicoccus undariae strain W-BA3 (KJ855315)
PMO118_11.3
0.050

f)

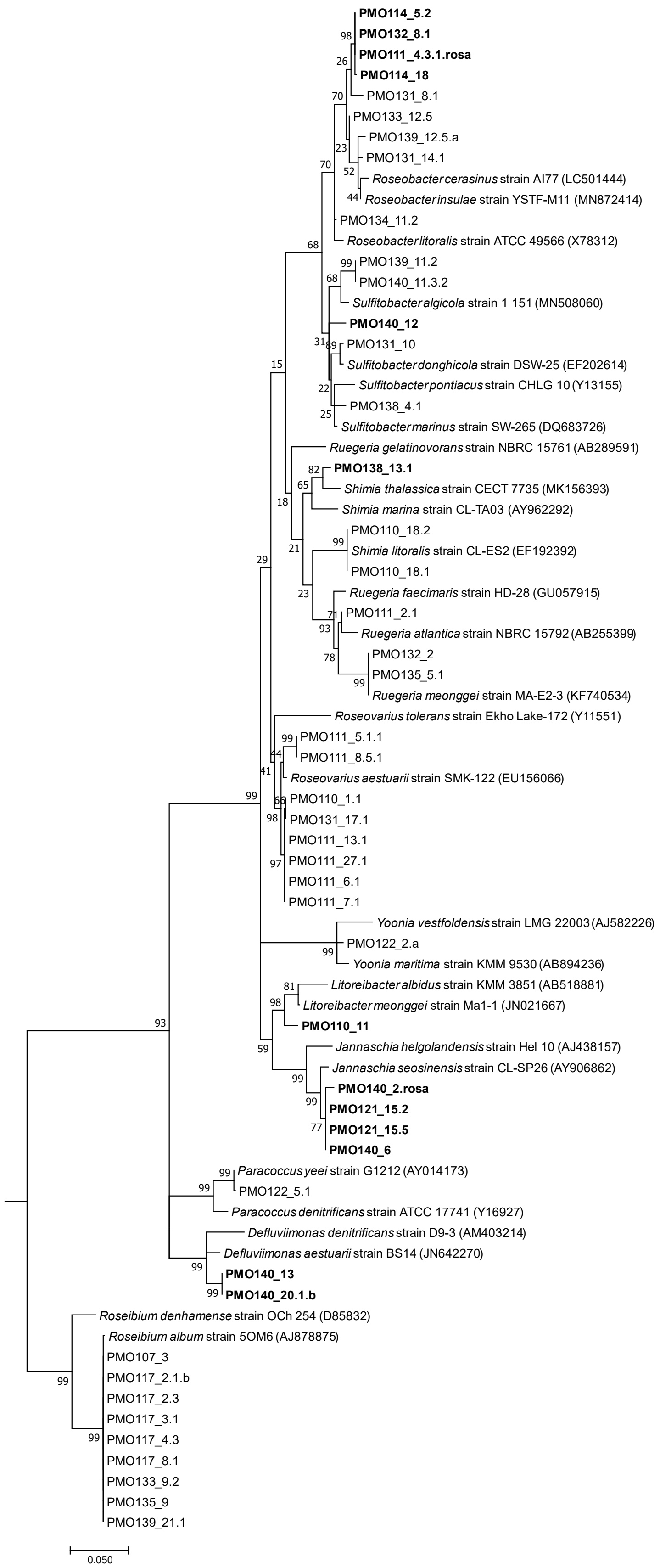

PMO114_5.2
PMO132_8.1
PMO111_4.3.1.rosa
PMO114_18
PMO131_8.1
PMO133_12.5
PMO139_12.5.a
PMO131_14.1
Roseobacter cerasinus strain AI77 (LC501444)
Roseobacter insulae strain YSTF-M11 (MN872414)
PMO134_11.2
Roseobacter litoralis strain ATCC 49566 (X78312)
PMO139_11.2
PMO140_11.3.2
Sulfitobacter algicola strain 1 151 (MN508060)
PMO140_12
PMO131_10
Sulfitobacter donghicola strain DSW-25 (EF202614)
Sulfitobacter pontiacus strain CHLG 10 (Y13155)
PMO138_4.1
Sulfitobacter marinus strain SW-265 (DQ683726)
Ruegeria gelatinovorans strain NBRC 15761 (AB289591)
PMO138_13.1
Shimia thalassica strain CECT 7735 (MK156393)
Shimia marina strain CL-TA03 (AY962292)
PMO110_18.2
Shimia litoralis strain CL-ES2 (EF192392)
PMO110_18.1
Ruegeria faecimaris strain HD-28 (GU057915)
PMO111_2.1
Ruegeria atlantica strain NBRC 15792 (AB255399)
PMO132_2
PMO135_5.1
Ruegeria meonggei strain MA-E2-3 (KF740534)
Roseovarius tolerans strain Ekho Lake-172 (Y11551)
PMO111_5.1.1
PMO111_8.5.1
Roseovarius aestuarii strain SMK-122 (EU156066)
PMO110_1.1
PMO131_17.1
PMO111_13.1
PMO111_27.1
PMO111_6.1
PMO111_7.1
Yoonia vestfoldensis strain LMG 22003 (AJ582226)
PMO122_2.a
Yoonia maritima strain KMM 9530 (AB894236)
Litoreibacter albidus strain KMM 3851 (AB518881)
Litoreibacter meonggei strain Ma1-1 (JN021667)
PMO110_11
Jannaschia helgolandensis strain Hel 10 (AJ438157)
Jannaschia seosinensis strain CL-SP26 (AY906862)
PMO140_2.rosa
PMO121_15.2
PMO121_15.5
PMO140_6
Paracoccus yeei strain G1212 (AY014173)
PMO122_5.1
Paracoccus denitrificans strain ATCC 17741 (Y16927)
Defluviimonas denitrificans strain D9-3 (AM403214)
Defluviimonas aestuarii strain BS14 (JN642270)
PMO140_13
PMO140_20.1.b
Roseibium denhamense strain OCh 254 (D85832)
Roseibium album strain 5OM6 (AJ878875)
PMO107_3
PMO117_2.1.b
PMO117_2.3
PMO117_3.1
PMO117_4.3
PMO117_8.1
PMO133_9.2
PMO135_9
PMO139_21.1
0.050

g)

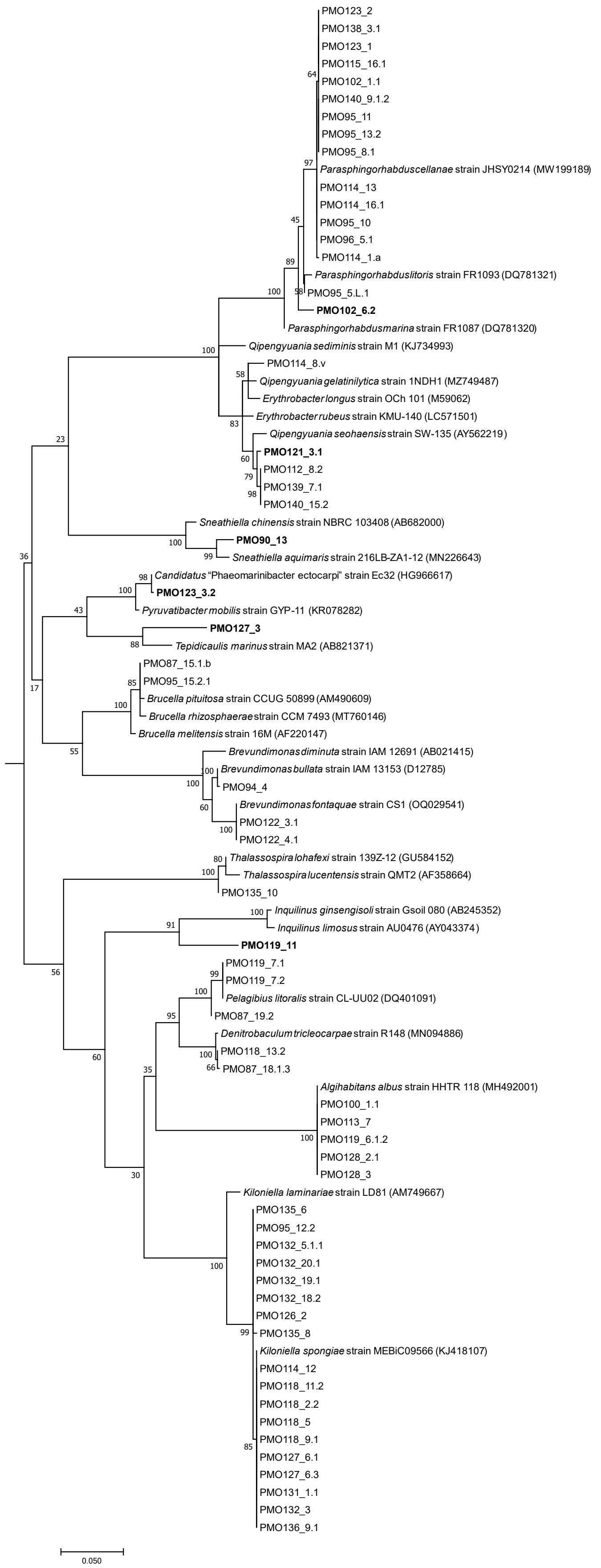

PMO123_2
PMO138_3.1
PMO123_1
PMO115_16.1
PMO102_1.1
PMO140_9.1.2
PMO95_11
PMO95_13.2
PMO95_8.1
Parasphingorhabduscellanae strain JHSY0214 (MW199189)
PMO114_13
PMO114_16.1
PMO95_10
PMO96_5.1
PMO114_1.a
Parasphingorhabduslitoris strain FR1093 (DQ781321)
PMO95_5.L.1
PMO102_6.2
Parasphingorhabdusmarina strain FR1087 (DQ781320)
Qipengyuania sediminis strain M1 (KJ734993)
PMO114_8.v
Qipengyuania gelatinilytica strain 1NDH1 (MZ749487)
Erythrobacter longus strain OCh 101 (M59062)
Erythrobacter rubeus strain KMU-140 (LC571501)
Qipengyuania seohaensis strain SW-135 (AY562219)
PMO121_3.1
PMO112_8.2
PMO139_7.1
PMO140_15.2
Sneathiella chinensis strain NBRC 103408 (AB682000)
PMO90_13
Sneathiella aquimaris strain 216LB-ZA1-12 (MN226643)
Candidatus "Phaeomarinibacter ectocarpi" strain Ec32 (HG966617)
PMO123_3.2
Pyruvatibacter mobilis strain GYP-11 (KR078282)
PMO127_3
Tepidicaulis marinus strain MA2 (AB821371)
PMO87_15.1.b
PMO95_15.2.1
Brucella pituitosa strain CCUG 50899 (AM490609)
Brucella rhizosphaerae strain CCM 7493 (MT760146)
Brucella melitensis strain 16M (AF220147)
Brevundimonas diminuta strain IAM 12691 (AB021415)
Brevundimonas bullata strain IAM 13153 (D12785)
PMO94_4
Brevundimonas fontaquae strain CS1 (OQ029541)
PMO122_3.1
PMO122_4.1
Thalassospira lohafexi strain 139Z-12 (GU584152)
Thalassospira lucentensis strain QMT2 (AF358664)
PMO135_10
Inquilinus ginsengisoli strain Gsoil 080 (AB245352)
Inquilinus limosus strain AU0476 (AY043374)
PMO119_11
PMO119_7.1
PMO119_7.2
Pelagibius litoralis strain CL-UU02 (DQ401091)
PMO87_19.2
Denitrobaculum tricleocarpae strain R148 (MN094886)
PMO118_13.2
PMO87_18.1.3
Algihabitans albus strain HHTR 118 (MH492001)
PMO100_1.1
PMO113_7
PMO119_6.1.2
PMO128_2.1
PMO128_3
Kiloniella laminariae strain LD81 (AM749667)
PMO135_6
PMO95_12.2
PMO132_5.1.1
PMO132_20.1
PMO132_19.1
PMO132_18.2
PMO126_2
PMO135_8
Kiloniella spongiae strain MEBiC09566 (KJ418107)
PMO114_12
PMO118_11.2
PMO118_2.2
PMO118_5
PMO118_9.1
PMO127_6.1
PMO127_6.3
PMO131_1.1
PMO132_3
PMO136_9.1
0.050

Supplementary Figure S1. Phylogenetic trees based on 16S rRNA gene sequences of isolates from Praia da Memória and their closest type strains. Trees correspond to isolates affiliated with different bacterial taxa:
(a) *Actinomycetota* [Outgroup not shown; *Actinomyces bovis* NCTC 11535 (accession number X81061)];
(b) *Planctomycetota* [Outgroup not shown; *Gemmata obscuriglobus* UQM 2246 (accession number X56305)];
(c) *Bacillota* [Outgroup not shown; *Bacteroides fragilis* ATCC 25285 (accession number AB042061)];
(d) *Bacteroidota* [Outgroup not shown; *Bacillus subtilis* IAM 12118 (accession number KP326374)];
(e) *Pseudomonadota* belonging to the classes *Betaproteobacteria* and *Gammaproteobacteria* [Outgroup not shown; *Myxococcus fulvus* NBRC 100333 (accession number AB218224)]; (f) *Pseudomonadota* (order *Rhodobacterales, class Alphaproteobacteria*) [Outgroup not shown; *Myxococcus fulvus* NBRC 100333 (accession number AB218224)]; and
(g) *Pseudomonadota* (orders *Caulobacterales, Hyphomicrobiales, Kiloniellales, Rhizobiales, Rhodospirillales, Sneathiellales*, and *Sphingomonadales, class Alphaproteobacteria*) [Outgroup not shown; *Myxococcus fulvus* NBRC 100333 (accession number AB218224)]. Phylogenetic trees were inferred using the Maximum Likelihood method under the General Time Reversible model with gamma-distributed rates and invariant sites (G+I). Bootstrap values (>50%) based on 1,000 replicates are shown at branch nodes. Evolutionary analyses were conducted in MEGA7. Putative novel taxa are presented in bold.
